# Supplementary material for: Transient chemical and structural changes in graphene oxide during ripening
Source: Nat Commun. 2024 Feb 24;15:1708. doi: 10.1038/s41467-024-46083-4 (PMC10894275; doi:10.1038/s41467-024-46083-4)
Supplement: Supplementary file 1 — Supplementary Information [file 41467_2024_46083_MOESM1_ESM.pdf]

Supplementary Information

Transient chemical and structural changes in graphene oxide during ripening

Hayato Otsuka,[1] Koki Urita,[2] Nobutaka Honma,[3] Takashi Kimuro,[4] Yasushi Amako,[5] Radovan Kukobat,[1,6] Teresa J. Bandosz,[7] Junzo Ukai,[3] Isamu Moriguchi,[2] and Katsumi Kaneko*[1]

[1]*Research Initiative for Supra-Materials, Shinshu University, 4-17-1 Wakasato, Nagano 380-8553, Japan*

[2]*Graduate School of Engineering, Nagasaki University, 1-14 Bunkyo-machi, Nagasaki, Nagasaki 852-8521, Japan*

[3]*New Material & Value Creation Gr., Project Material Creation Dept., Mobility Material Engineering Div., Toyota Motor Corporation, 1, Toyota-cho, Toyota, Aichi, 471-8572, Japan*

[4]*Development Gr.2, Development Section, Engineering Dept., Sanwayuka Industry Corporation, Fukada 15, Ichiriyamacho, Kariya, Aichi, 448-0002, Japan*

[5]*Department of Physics, Faculty of Science, Shinshu University, 3-1-1 Asahi, Matsumoto, Nagano, 390-8621, Japan*

[6]*Department of Chemical Engineering and Technology, Faculty of Technology, University of Banja Luka, B.V. Stepe Stepanovica 73, 78 000 Banja Luka, Bosnia and Herzegovina*

[7]*Department of Chemistry and Biochemistry, The City College of New York, 160 Convent Avenue, New York, NY 10031, United States*

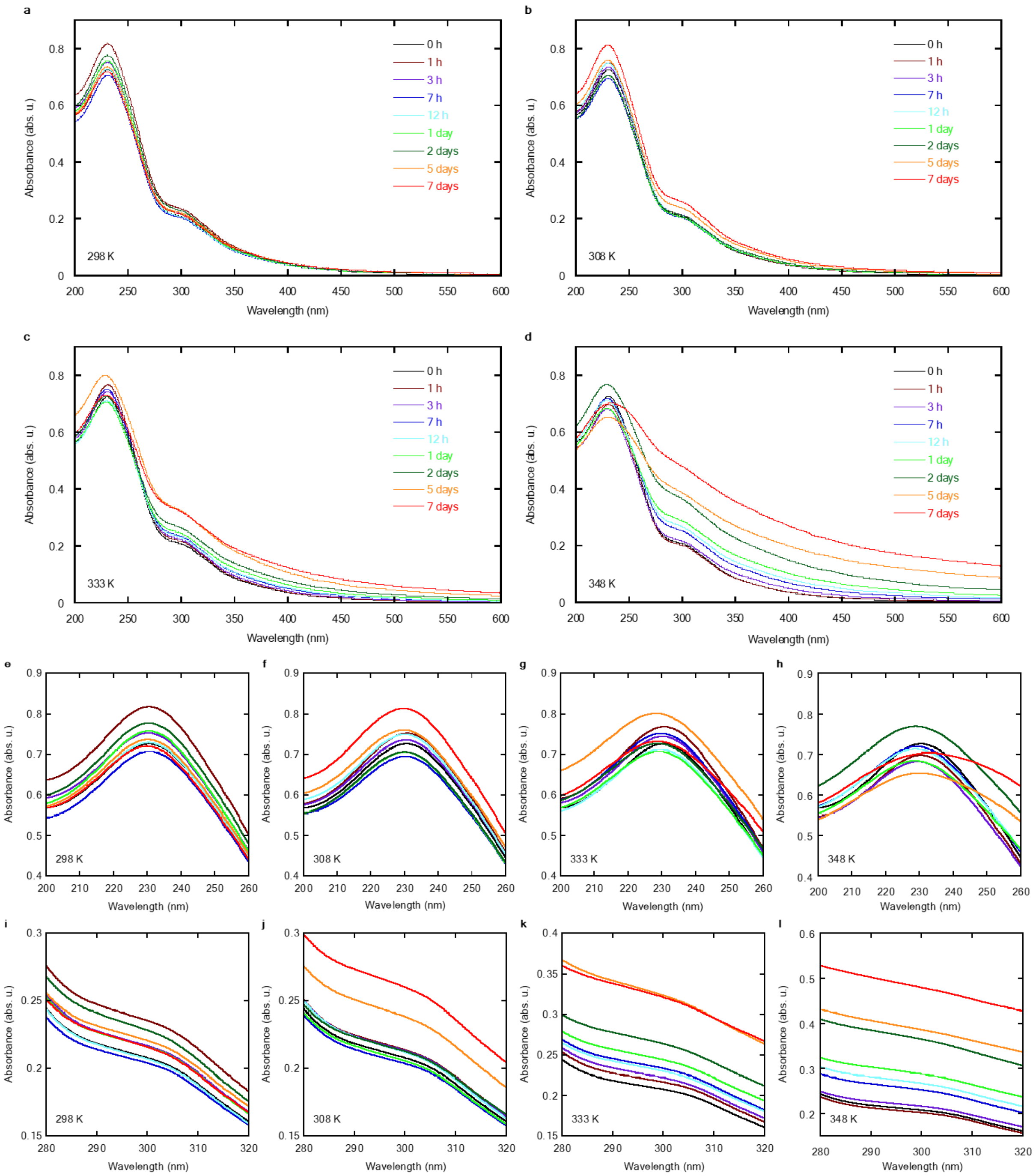

Supplementary Fig. 1 | Changes in the UV-Vis spectra for the GO colloids as a function of the ripening time at different temperatures. a–d Full range of the UV-Vis spectra. **e–h** Magnified spectra in the range of 200-260 nm. **i–l** Magnified spectra in the range of 280-300 nm. The ripening time: black, 0 h; brown, 1 h; purple, 3 h; blue, 7 h; pale blue, 12 h; yellow-green, 1 day; green, 2 days; orange, 5 days and red, 7 days.

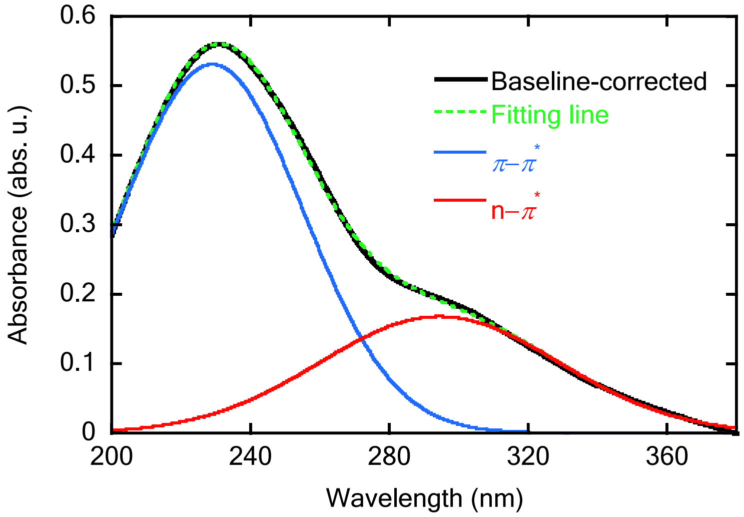

Supplementary Fig. 2 | Example of peak deconvolution of the UV-Vis spectrum of the GO colloid ripened at 348 K for 2 days.

Supplementary Note 1 | Definition and significance of reduced time.

We introduce the reduced time to describe ripening processes at different temperatures based on a definite energy level as the standard. The peak position of the $\pi - \pi^*$ transition band depends on the ripening time and temperature. If the ripening processes at different temperatures proceed in the same way we can describe the time changes of the peak position at different temperatures with a single relation by introducing the optimum reduced time (t_{red}). The peak position varies from 230.4 to 228.8 nm for UV-Vis spectra of iGO and mGO. We select the intermediate energy level (229.6 nm) between these positions as the standard energy level. Then, we determine the ripening time for shifting of the peak position to 229.6 nm at different temperatures as follows. The times for shifting to 229.6 nm are 336 h at 298 K, 168 h at 308 K, 12h at 333 K, and 3 h at 348 K. The reduced time at different temperature is obtained, as given below.

$$t_{\mathrm{red}}(298\ \mathrm{K}) = t_{298} \times \frac{3\ \mathrm{h}}{336\ \mathrm{h}} = \frac{1}{112} t_{298} \quad (1)$$

$$t_{\mathrm{red}}(308\ \mathrm{K}) = t_{308} \times \frac{3\ \mathrm{h}}{168\ \mathrm{h}} = \frac{1}{56} t_{308} \quad (2)$$

$$t_{\mathrm{red}}(333\ \mathrm{K}) = t_{333} \times \frac{3\ \mathrm{h}}{12\ \mathrm{h}} = \frac{1}{4} t_{333} \quad (3)$$

$$t_{\mathrm{red}}(348\ \mathrm{K}) = t_{348} \times \frac{3\ \mathrm{h}}{3\ \mathrm{h}} = t_{348} \quad (4)$$

Here, $t_{T\ \mathrm{K}^{-1}}$ represents the ripening time at each temperature.

Supplementary Table 1 | Lifetimes of iGO and mGO at different ripening temperatures.

Temperature	298 K	308 K	333 K	348 K
iGO	112 h	56 h	4 h	1 h
mGO	190 days	90 days	7 days	2 days

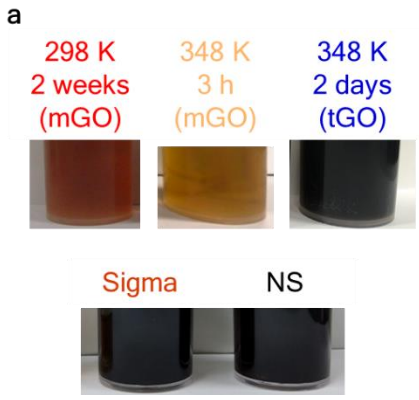

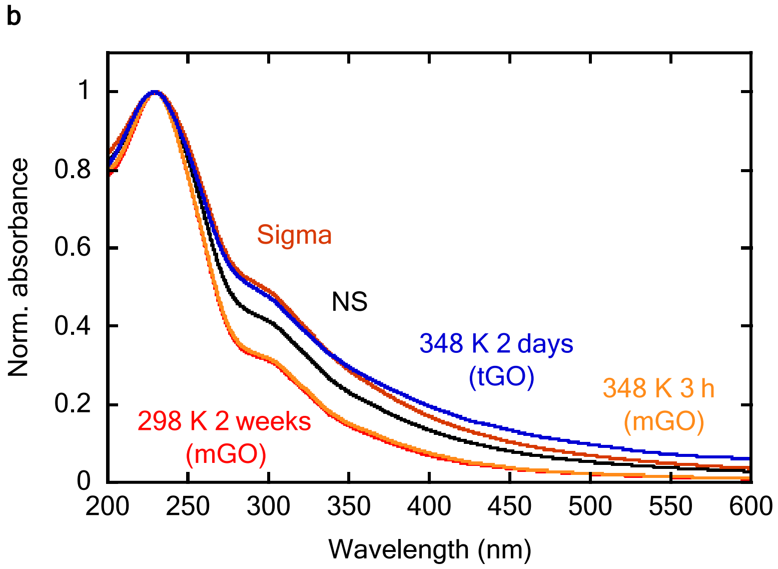

Supplementary Fig. 3 | Comparison of the GO colloids ripened in this study with commercial ones. a Colors of the ripened GO colloids (upper panels) and commercial GO colloids (bottom). The light-colored GO colloids ripened correspond to the mGO and black one to the tGO. **b** UV-Vis spectra of the commercial GO colloids and three ripened GO colloids whose $\pi - \pi^*$ transition peak positions are at 229.6 nm. The absorbance is normalized by the peak intensity of the $\pi - \pi^*$ transition peak. GO: Red, GO ripened at 298 K for 2 weeks; orange, GO ripened at 348 K for 3 h; blue, GO ripened at 348 K for 2 days; brown, GO colloid purchased from Sigma-Aldrich Co., LLC (Sigma); black, GO colloid purchased from Nippon Shokubai Co., Ltd. (NS).

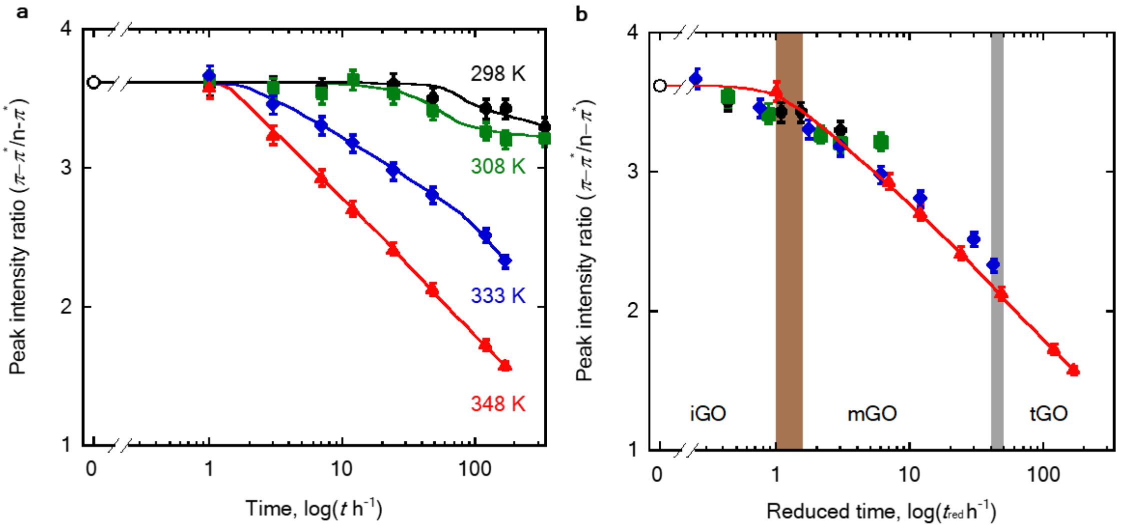

Supplementary Fig. 4 | Changes of peak intensity ratio of the $\pi-\pi^*$ and n $-\pi^*$ transitions upon ripening. a Changes with the logarithmic scale of ripening time. **b** Changes with the reduced time (t_{red}) used in Fig. 3. Brown and gray zones represent the transition regions. Ripening temperature: black solid line and circles, 298 K; green solid line and squares, 308 K; blue solid line and diamonds, 333 K; red solid line and triangles, 348 K. The peak ratio of the non-ripened GO colloids is shown with open circles. The error bars represent the reproducibility in the peak position determination of at least three measurements.

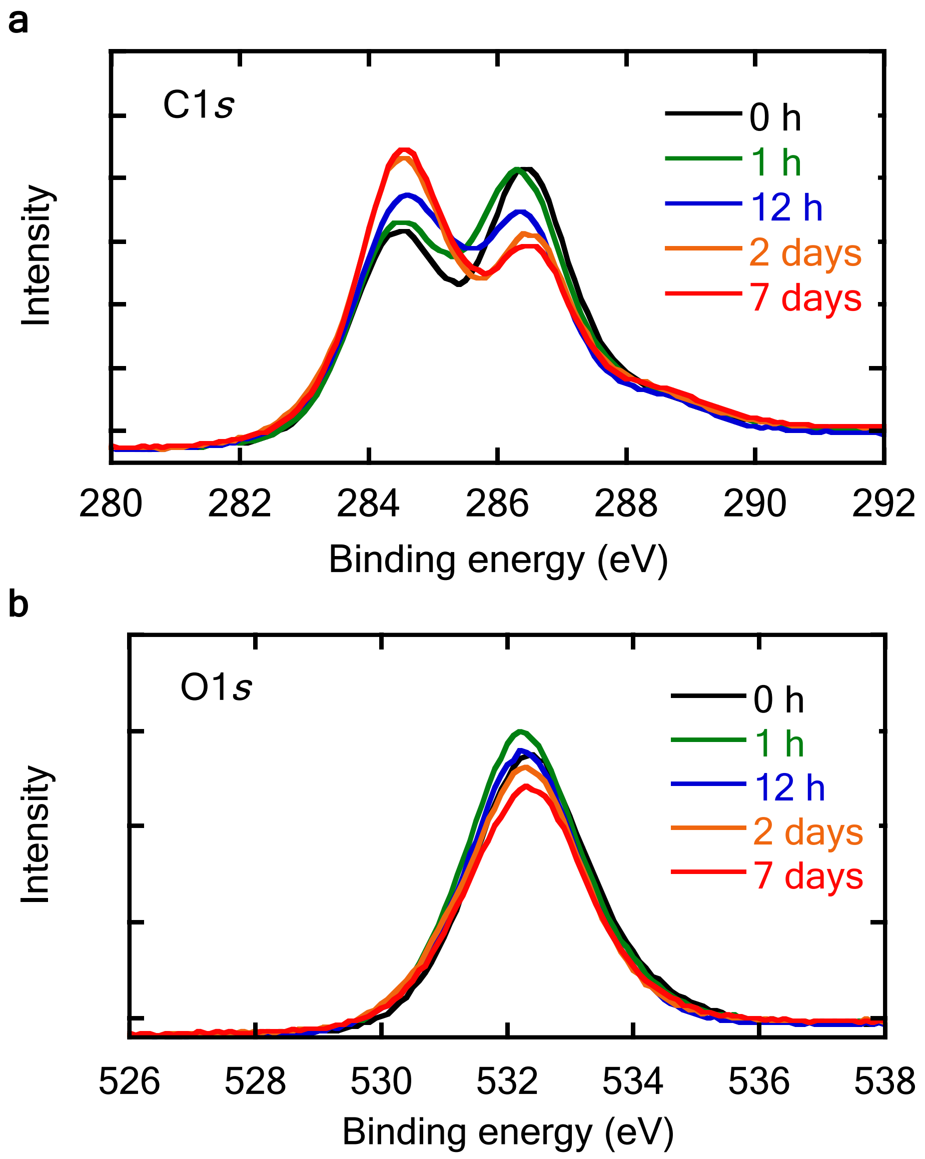

Supplementary Fig. 5 | XPS spectra of the freeze-dried GO ripened for different durations at 348 K. a C1*s*. **b** O1*s*.

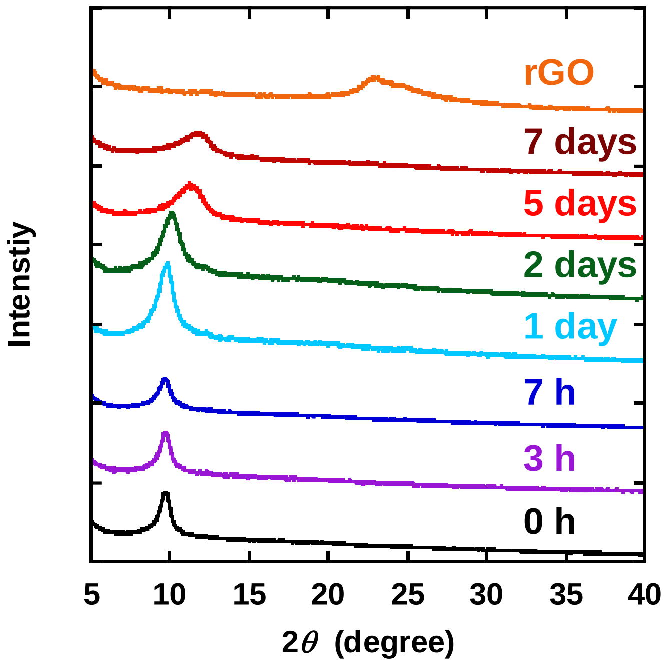

Supplementary Fig. 6 | XRD patterns of the freeze-dried GO ripened for different durations at 348 K and rGO.

Supplementary Note 2 | Determination and terminology of thickness along stacking direction. Graphene oxide has the unit structure consisting of several oxidized graphene-like sheets. The thickness of the stacking sheets along the *c*-axis is called the "stack height". This expression is often used for micrographite unit structures of disordered carbon. The stack height is expressed as L_c. In this paper, we use the average number of stacking layers instead of the stack height for showing the stacking structure more clearly. The average number of stacking layers is calculated as follows

$$\text{Average number of stacking layers} = \frac{L_c}{d_{001}} + 1 \tag{5}$$

Lc is determined from the Scherrer equation using the full width half maximum (FWHM) of the 001 peak of GO and d_{001}, i.e. the interlayer distance of the (001) planes based on the Bragg equation. The broadening of the peak caused by instruments is corrected by subtracting the FWHM of the graphite 002 peak, which has a sufficient crystallite size.

$$\mathrm{FWHM_{GO-001}} = \sqrt{(\mathrm{FWHM^2_{GO-001-observed}} - \mathrm{FWHM^2_{Graphite-002}}} \tag{6}$$

$$Lc = 0.94 \times \frac{0.15418}{\frac{\mathrm{FWHM_{GO-001}}}{180}\pi \times \cos\left(\frac{2\theta}{360}\pi\right)} \tag{7}$$

Here, the 001 peaks of most of the samples are not of λ–shape. Then, we use the shape factor of 0.94, which is the shape factor for the three–dimensional structure. The wavelength of the X-rays is 0.15418 nm and θ denotes the position of the 001 peak.

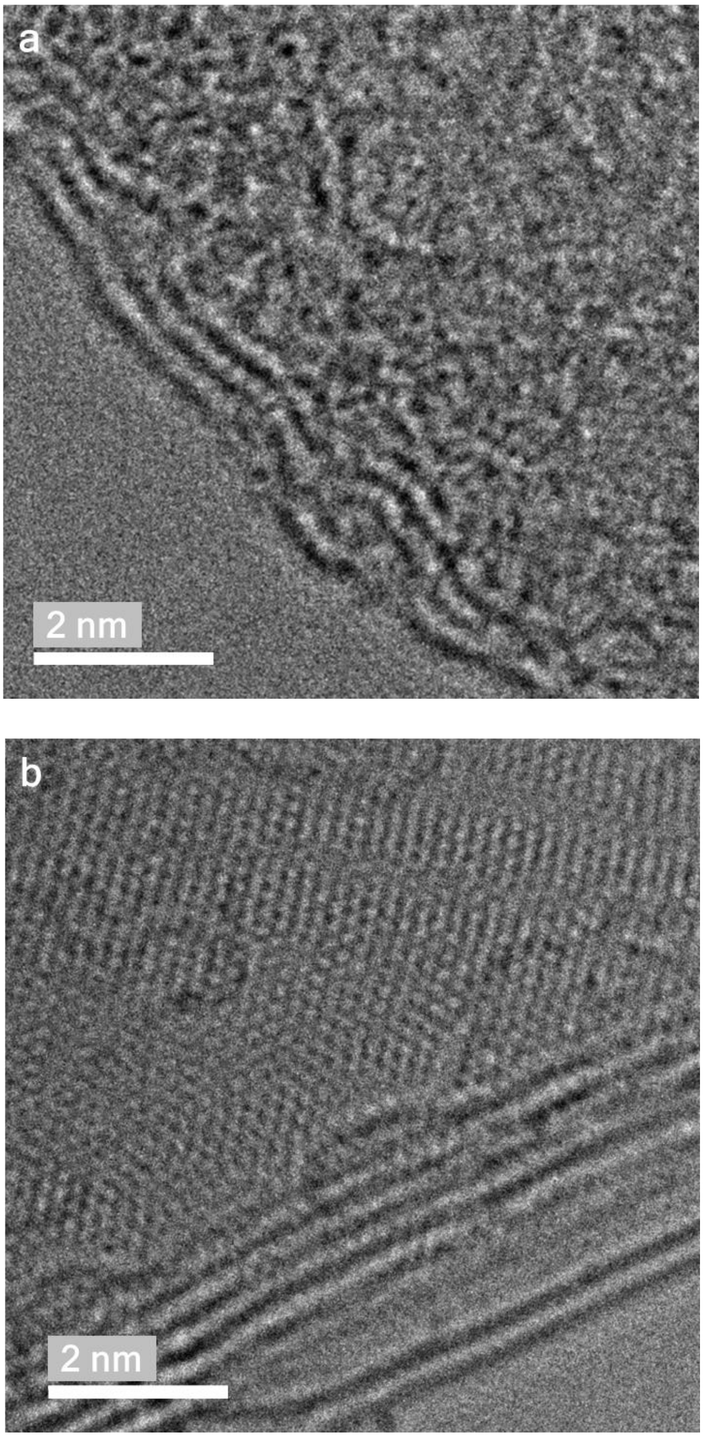

Supplementary Fig. 7 | Effect of annealing at 2073 K in Ar on the structure of GO. a Non-annealed sample. **b** Annealed sample.

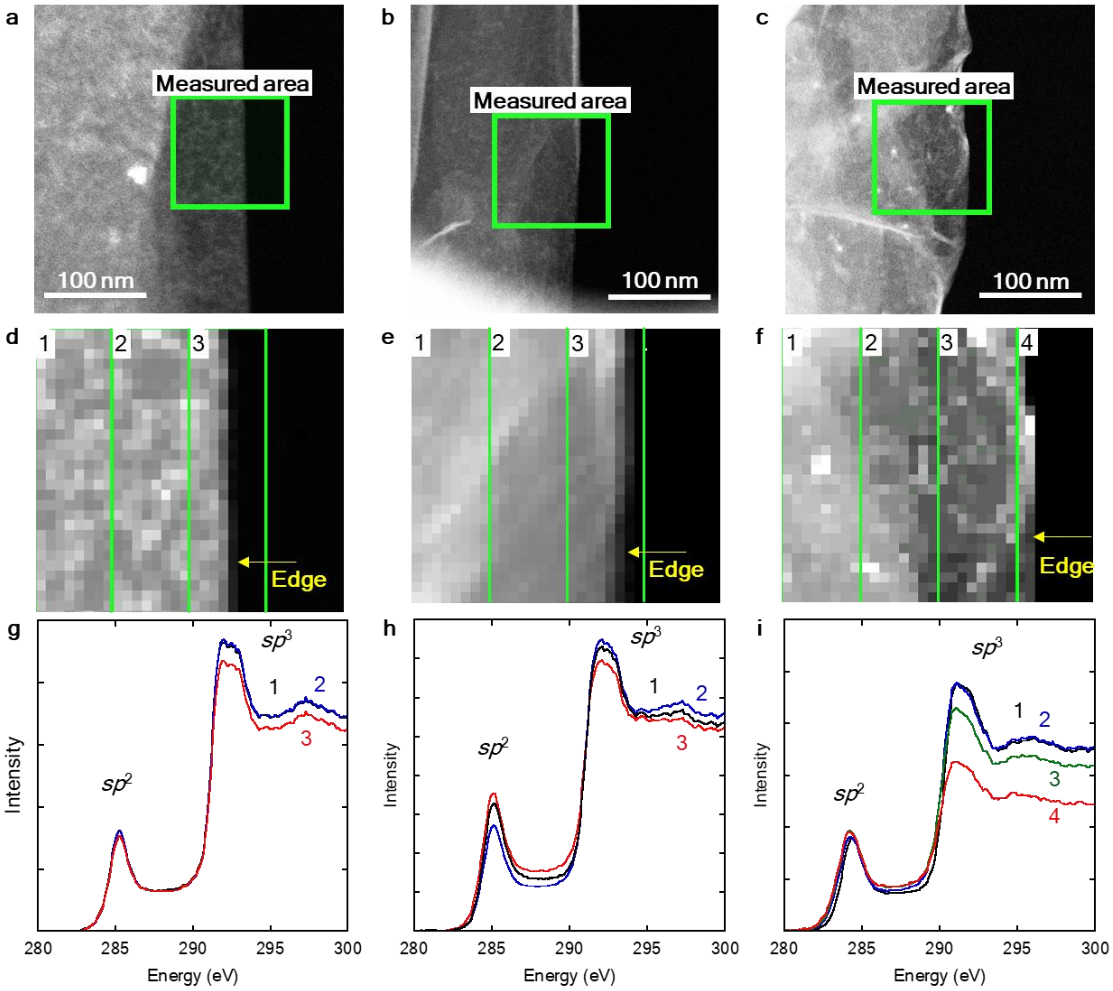

Supplementary Fig. 8 | TEM images and EELS spectra of the freeze-dried GO powder annealed in Ar at 2073 K. **a–c** TEM images of the freeze-dried GO powder annealed in Ar at 2073 K. The EELS spectra were measured on the green marked region in the TEM images. **d–f** TEM images of green marked region in **a–c**. The images are divided into 4 parts parallel to the edge and each part is numbered from 1 towards the edge. The boundaries are indicated by green lines. **g–i** The EELS spectra of each position shown in **d–f**. **a**, **d, g** Non-ripened GO. **b**, **e**, **h** GO ripened at 348 K for 2 days. **c**, **f**, **i** GO ripened at 348 K for 7 days.

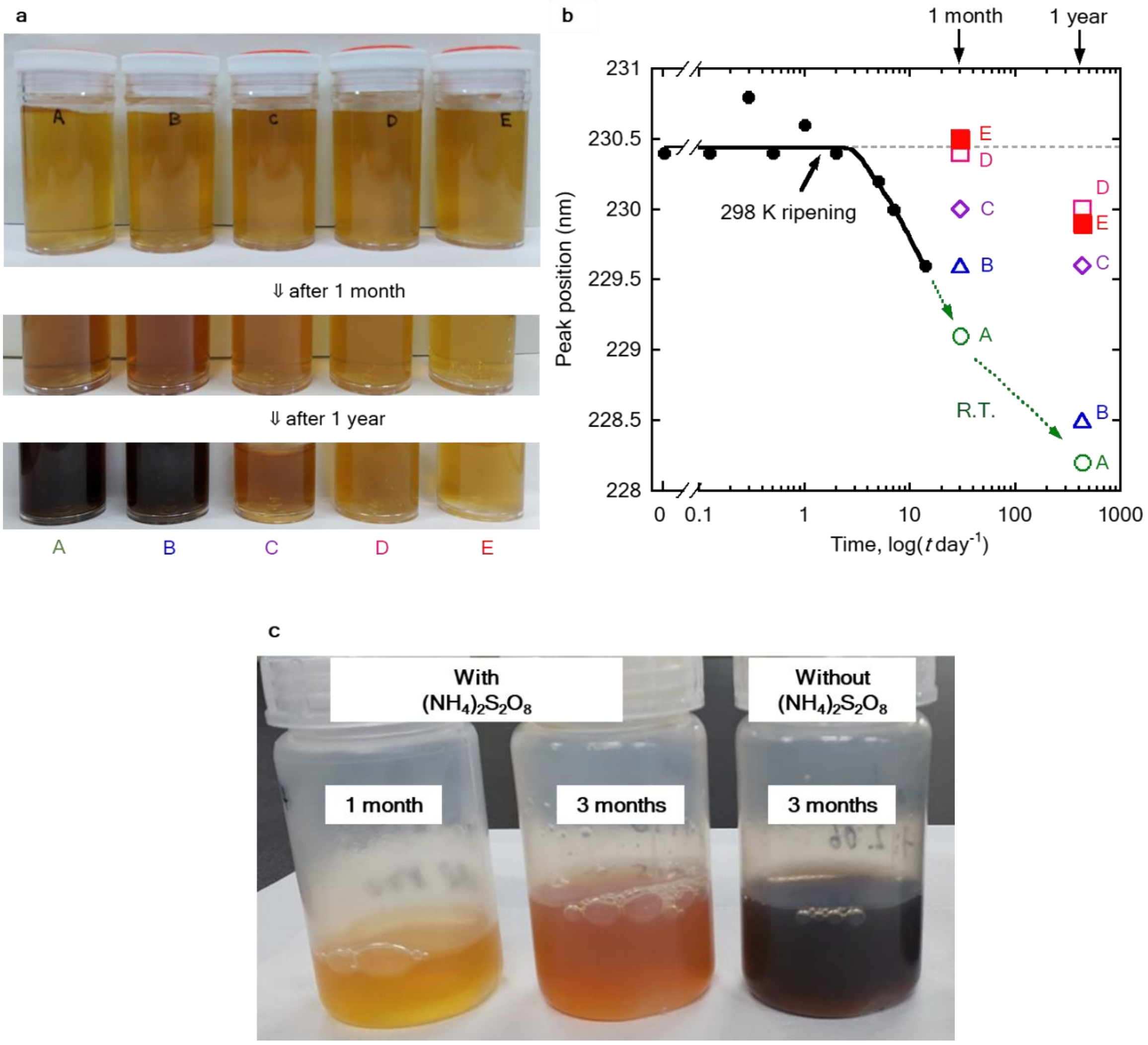

Supplementary Fig. 9 | Color changes of the colloidal dispersion and change in the peak position of the $\pi - \pi^*$ transition. a Color changes under different storage condition. A: Room temperature (R.T.), B: Shielding light under R.T., C: Stored at 280 ± 2 K in a refrigerator, D: Stored at 255 ± 2 K in a freezer, E: Stored at 255 ± 2 K in a freezer after being instantaneously frozen with liquid nitrogen. **b** Changes in the peak positions of the $\pi - \pi^*$ transition. Gray dashed line shows the peak position of the $\pi - \pi^*$ transition for iGO. **c** Color change of the GO colloid (0.1 wt%) with and without 1 wt% ammonium peroxydisulfate.

Supplementary Note 3 | Classification of three GO states.

The range of the peak position of the $\pi-\pi^*$ transition for tGO includes those for iGO and mGO owing to the diverse changes in the peak position of the $\pi-\pi^*$ transition, although the color of tGO differs significantly from those of iGO and mGO (Fig. 3 and Supplementary Fig. 3). This is partially attributed to the low reliability of determination of the peak position, since the peaks of the $\pi-\pi^*$ and $\mathrm{n}-\pi^*$ transitions overlap considerably. The peak position of the $\pi-\pi^*$ transition is not applicable for the clear classification of tGO. Therefore, comparative characterization with XPS, XRD analysis, TEM and measurements of magnetic and electrical properties, in addition to UV-Vis spectroscopic investigation can provide the most reliable classification of iGO, mGO and tGO.
